# Comprehensive scoping review of palliative care development in Africa: recent advances and persistent gaps

**DOI:** 10.3389/frhs.2024.1425353

**Published:** 2024-12-09

**Authors:** Fernanda Bastos, Eduardo Garralda, Alvaro Montero, John Y. Rhee, Natalia Arias-Casais, Emmanuel Luyirika, Eve Namisango, José Pereira, Carlos Centeno, Vilma A. Tripodoro

**Affiliations:** ^1^ Institute for Culture and Society, ATLANTES Global Observatory of Palliative Care, University of Navarra, Pamplona, Spain; ^2^ IdiSNA, Navarrese Centre for Sanitary Research, Pamplona, Spain; ^3^ Division of Adult Palliative Care, Department of Supportive Oncology, Dana-Farber Cancer Institute, Harvard Medical School, Boston, MA, United States; ^4^ Center for Neuro-Oncology, Department of Medical Oncology, Dana-Farber Cancer Institute, Harvard Medical School, Boston, MA, United States; ^5^ Department of Paediatrics, Elbekliniken Stade, Stade, Germany; ^6^ African Palliative Care Association, Kampala, Uganda; ^7^ Faculty of Medicine, University of Navarra, Pamplona, Spain; ^8^ Division of Palliative Medicine, Department of Family Medicine, McMaster University, Hamilton, ON, Canada

**Keywords:** Africa, palliative care, health services, education, health policies, medicines, palliative care development

## Abstract

Worldwide 56·8 million people need palliative care (PC), and Africa shows the highest demand. This study updates the 2017 review of African PC development, using a scoping review methodology based on Arksey and O'Malley's framework and the PRISMA-ScR checklist. The review was conducted across PUBMED, CINAHL, Embase, government websites, and the African PC Association Atlas, from 2017 to 2023, charting its progress using the new WHO framework for PC Development, which, in addition to Services, Education, Medicines, and Policies, two new dimensions were incorporated: Research and Empowerment of people and Communities. Of the 4.420 records, 118 met the inclusion criteria. Findings showed increased adult specialised services (*n* = 675), and 15 of 54 countries have paediatric services. Nonetheless, the ratio of services per population mostly remains under 0,10 per 100.000 inhabitants. PC education was included in undergraduate curricula in 29 countries; despite the rise in morphine availability (28 countries), median consumption remains under 3 mg/per capita/year, and 14 countries presented stand-alone policies. Publications on PC development increased, and 26 countries have National PC Associations. Notwithstanding progress since 2017, significant hurdles remain, highlighting the need for ongoing research and policy development to ensure equitable access to palliative care in Africa.

## Background

In 2017, a review by Rhee et al. (1) found that palliative care (PC) services were documented in only 19 (35%) African countries, with Kenya, South Africa, Tanzania, Uganda, and Zimbabwe leading in service provision. However, despite Uganda being among the top five, only 10% of its palliative care needs are met (2). Also, Africa is estimated to represent 51,8% of the world´s children's PC needs (3). Besides the burden of suffering, this has other implications such as access to food, school fees, shelter, orphan care, income generation, transportation to facilities, and funeral costs. This shortfall is particularly alarming given that Africa is projected to see a 126% increase in this burden by 2060 (4).

These statistics underscore the urgent need to expand and improve PC across the continent, especially as it has been shown as a cost-effective approach to relieve serious health related suffering, including in Low- and Middle-Income Countries (5, 6).

While there are multiple reports in the literature related to PC development in Africa, these tend to be specific to one country or small groupings of countries and focus on one or other aspects of PC. Fewer have assessed the status of PC more broadly across all African countries and various domains. Clark and colleagues published one of the first such studies in 2007 (7). In 2015, the Economist's Intelligence Unit published an international report. Still, it included only 13 (24%) African countries (8), and the subsequent cross-country assessment by Finkelstein et al. included only eight (15%) African countries among the 81 countries studied (9). Although the analysis included some dimensions of PC development, the main focus was on the quality of death and dying. The scoping review by Rhee et al. in 2017 (1), which focused on the domains of the Public Health Strategy for PC (10), identified activity across 26 of Africa's 54 countries, and in the same year, the “APCA (African PC Association) Atlas of PC in Africa” was published with information of 48 African countries (11). Previous studies have shown that despite numerous obstacles, progress in PC development has been noted over the last decade (1, 6). This has included areas such as integrating PC services as part of the public health care systems and integrating PC into educational curricula. Despite access to opioids and morphine being a common barrier, enhancing access to morphine has been observed in countries such as Kenya, Rwanda, South Africa, Tanzania and Uganda (5, 6). They also reported that most services were concentrated in Kenya, South Africa, and Uganda; and stand-alone policies existed in only seven countries. Despite a paucity of development in most African countries, there were some encouraging signals of capacity-building across the continent (1).

Since the 2017 studies (1), the Declaration of Astana, in 2018, renewed the political commitment to integrating PC into the health system as part of Universal Health Coverage (Sustainable Development Goals target 3.8) (12). And the COVID-19 pandemic (2020 to 2023) also drew attention to palliative care. Our hypothesis is that since 2017, noteworthy development has occurred in palliative care development on the African continent.

The goal of our study is to update the 2017 report incorporating the WHO's new framework for PC development (published in 2021) (13), which includes two new dimensions, namely empowering people and communities and research, to the four dimensions of WHO's Public Health Strategy for integration of PC (health policies, education and training, use of essential medicines and provision of PC integrated health services) (10).

## Methods

A scoping review approach was selected over the systematic or other forms of reviews as this most closely aligned with our study goals to identify the nature and extent and characterise the quantity and quality, of the germane literature. The Arksey and O'Malley methodology (14) and the Preferred Reporting Items for Systematic Reviews and Meta-Analyses extension for Scoping Reviews (PRISMA-ScR) checklist (15) were followed.

### Conceptual framework

The WHO's new consensus-derived conceptual model, which describes the core components required for PC development in a country, informed our review. The model comprises six assessment components: (i.) appropriate health policies; (ii.) empowering people and community involvement (iii.) palliative care-related research (iv) adequate access to medicines; (v.) education of healthcare workers and the public; (vi.) implementation of palliative care services at all levels of the health system (13).

### Search strategy and selection criteria

We searched the PubMed, Embase and CINAHL databases using the “specific search filters” for PC validated by Rietjens et al. for each of the 54 African countries (See Table 1) (16).

**Table 1:** Search strategy.

PubMed	(“Terminal Care”(mh) OR bereave* OR hospice*(tw) OR “advanced cancer”(tiab) OR “end of life” OR “terminally ill”(tw) OR palliative*(tiab) OR “Palliative Care”(mh))	AND (Country)	AND (2017–2023
Embase	(“Terminal Care”/exp OR bereave* OR hospice*:de,it,lnk,ab,ti OR “advanced cancer”:ab,ti OR “end of life” OR “terminally ill”:de,it,lnk,ab,ti OR palliative*:ab,ti OR “palliative therapy”/exp)	AND (Country)	AND (2017–2023
CINAHL	(mh Terminal Care + OR bereave* OR hospice* OR “advanced cancer” OR “end of life” OR “terminally ill” OR palliative* OR mh Palliative Care OR mh palliative therapy+)	AND (Country)	AND (2017–2023

The specific search terms used for each database as validated by Rietjens et al. (16).

The following constituted the inclusion criteria: published in peer-reviewed journals; since the last comprehensive review was in 2017, we focused on data from January 2017 to March 2023 inclusive; covering at least one dimension of palliative care development according to the new WHO framework (e.g., empowering of people and communities, policy, research, education, essential medicines, services provision) (13); national-level data or findings; and any language. A broad spectrum of article types was allowed, including qualitative and quantitative studies, conference abstracts, published conference presentations, letters to editors, opinion papers, commentaries and editorials (see Table 2). Exclusion criteria included clinical trials; disease-specific articles (e.g., PC in cervical cancer), articles not specific to the country of search, articles that did not describe an aspect of PC development, and publications before 2017.

**Table 2:** Inclusion criteria.

Category	Criteria
Publication type	Published in peer-reviewed journals
Publication date range	From January 2017 to March 2023, inclusive
Article types	Qualitative and quantitative studies Conference abstracts and presentations Letters to editors Opinion papers Commentaries and editorials
WHO framework components	Empowering of People and Communities
	Policy
	Research
	Education
	Essential Medicines
	Service Provision
Scope of the findings	National-Level
Language	Any language

### Additional information sources

In addition to the databases, we also searched targeted websites in a country if two or fewer published articles met the inclusion criteria for that country. The websites included the country's Ministry of Health and/or other official government websites. The APCA Atlas of PC in Africa (11) served as a baseline information source.

### Data management

We compiled all retrieved articles into individual Google Sheets^Ⓡ^, one for each country. Duplicates of articles across search engines were then removed. Next, a decision on whether to include each article was reached by consensus between pairs of authors (AM, FB, EG, NA, VT) by reviewing each identified article's title, abstract, and full text. In cases where there was disagreement between the pairs, a third author was consulted. The extracted information concerned at least one dimension of the new WHO conceptual framework (13) (See Supplementary Table S1), which was then recorded into spreadsheets, one for each country.

This research is part of a larger project approved by the Ethical Board of the University of Navarra (Spain), registered under the number 2023.055.

## Results

**Figure 1:**
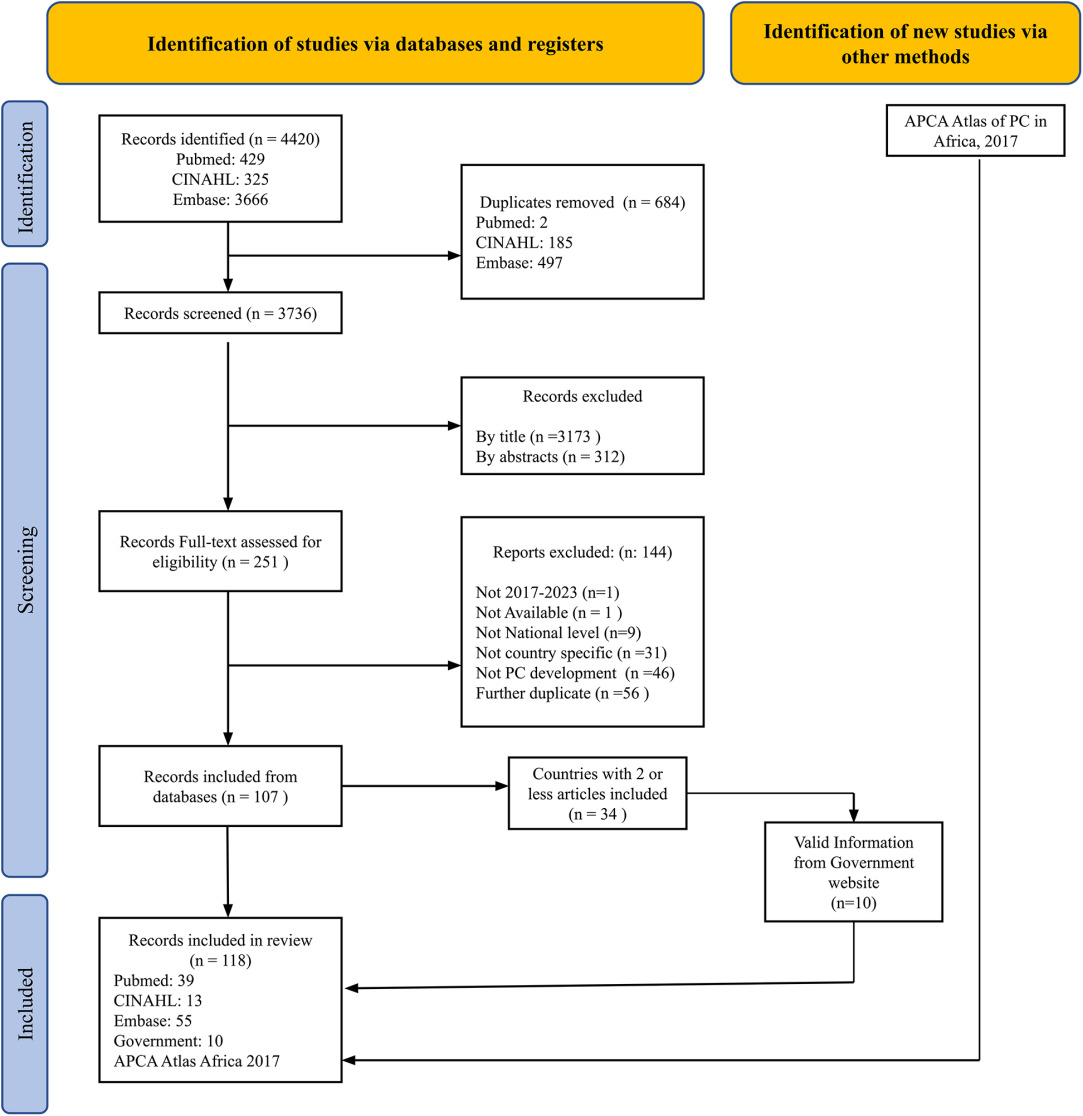
Scoping review study selection (PRISMA-flow diagram).

A total of 4,420 articles were identified in the initial search, and 684 duplicate articles were removed. 3,736 records were screened, 3,172 were excluded by title, and 312 by abstracts. Of the 251 potentially eligible articles, 144 were discarded following an assessment of their full text. Of the 34 countries with two or fewer articles, ten had information germane to this review on the targeted websites. A total of 118 records (107 from the database search, 11 from other sources and APCA Atlas of Africa) were included in the final analysis (See PRISMA flow diagram, Figure 1).

The key findings extracted from these articles are organised according to the WHO's new conceptual framework for PC development (13) and summarised in Table 3.

**Table 3:** Summary of Key findings across core dimensions.

WHO framework dimension	Key findings
Empowering people and communities	• National Associations of Palliative Care were reported in 48% of the countries (*n* = 26/54) (1, 11, 17) • Volunteer programs were identified in 15% of the countries (*n* = 8/54) (18–21) • Strategies to raise PC awareness found in 15% of the countries (*n* = 8/54) (6, 17, 18, 22–24)
Health policies	• Standalone national palliative care policies were found in 26% of countries (*n* = 14/54) (1, 6, 17, 22, 25–33) • PC office desk on Ministry of Health were reported in 39% of countries. (*n* = 21/54) (6, 17, 34) • Integration of PC in Primary health care systems are minimum. (*n* = 3/54) (6)
Research	• National Research groups on palliative care were identified in 5% of countries (*n* = 3/54) (6, 22, 34, 35) • National Congresses or conferences were reported in 15% of countries. (*n* = 8/54) (17) • PC development articles retrieved for 54% of countries (*n* = 29/54)
Education	• Palliative care was integrated into undergraduate medical or nursing curricula in 44%of countries. (*n* = 24/54) (11, 17, 31, 34, 36, 37) • Postgraduate programs found in 15% of countries. (*n* = 8/54) (1, 11, 25, 28, 38–44) • Paediatric PC education programs were reported in 7% of countries (*n* = 4/54) (1, 37, 41)
Essential medicines	• Morphine were available in 52% of countries. (*n* = 28/54) (6, 17, 22, 25, 36, 45–48) • National production of oral morphine was reported in 13% of countries.(*n* = 7/54) (6, 11, 19, 22, 25, 49) • Nurses or midwives are allow to prescribe opioid in 20% of countries. (*n* = 11/54) (17, 29, 50)
Service provision	• Palliative care services reported in 68% countries, in total 675 services were identified. (*n* = 37/54) (2, 6, 11, 17, 26, 31, 33, 34, 40, 42, 48, 51–54) • About 70% of all services in the continent are concentrated in only three countries: Uganda, Kenya and South (2, 6, 51) • Paediatric services reported in 28% of countries. (*n* = 15/54) (1, 11, 26, 34, 55)

### Empowerment of people and communities

National Associations of PC were founded in 26 countries (Table 4) (1, 11, 17). Diverse ways of cooperation were identified in ten countries. These included support for legal services to patients in Kenya (6, 22) and Uganda (6, 25); Civil Societies in Algeria, Tunisia and Morocco (35), engagement of traditional healers in Cameroon (55) and Mozambique (38), and collaboration with non-governmental organisations (NGOs) in the Democratic Republic of Congo (56) (hereafter DRCongo), Ghana (57), Uganda (25) and Rwanda (6).

**Table 4:** Countries with national association of PC (5, 15, 16).

Benin^a^ Botswana^a^ Burundi^a^ Cameroon^a^ Congo^a^ Côte d'Ivoire	DRCongo^a^ Gambia, The^a^ Ghana^a^ Guinea^a^ Kenya	Malawi Morocco Mozambique Namibia^b^ Nigeria Rwanda	Senegal^a^ Sierra Leone^a^ South Africa Tanzania	Togo^a^ Tunisia^a^ Uganda Zambia Zimbabwe

Twenty-six countries with established National Palliative Care Associations, highlighting new associations since the Rhee et al. review.

^a^ New associations since Rhee et al. (5).

^b^ APCA office functions as the national association (16).

Initiatives related to advance care planning were identified in Uganda, Sudan and South Sudan, mainly in national guidelines related to COVID-19 clinical management (58).

General PC awareness was low among health professionals in Botswana (26), Egypt (59), Eswatini (27), Nigeria (60), and Uganda (22). Public knowledge and understanding of PC were rated medium in South Africa (61) and Egypt (34). Strategies to raise awareness of PC were found in eight countries. Media outreach campaigns were described in Kenya, Uganda (6, 22, 23), Rwanda and Zambia (6, 23). Training programs targeting health care workers, journalists and community members were reported in Sao Tome and Principe (24), Mozambique, Sudan (17), Kenya, Rwanda (6, 23), Uganda (22) and Zambia (23). A national colloquium on PC in Madagascar, the first in that country, contributed to advancing awareness in Madagascar (18).

Health workforce shortages in PC generally are reported across Africa (38, 45, 46, 61, 62). In Liberia (63) and South Africa (61) some hospices rely mainly on volunteer staff. In Botswana, Egypt, Uganda (64), Malawi (39) and Sudan (46) a variety of volunteer-driven work was also described. For instance, in Botswana, volunteers are usually over 50 years old and in Uganda under 30 (64). In Liberia, nurses without PC training volunteer to provide care to patients and support to their caregivers at home (65). In Sudan, volunteers include health professionals with PC degrees (46). In Malawi, relatives care for patients in hospitals (62) and in some rural areas community health workers volunteer to provide rehabilitation care (66). In Uganda, a program in a community is described in which a multidisciplinary team of volunteers recruited through faith groups work as community consultants finding cases, and providing social support and end-of-life care (64).

### Health policies

The inclusion of PC in health policy-related national plans is listed in Table 5, divided into the following categories: standalone national PC policies, formal national cancer or HIV policies addressing PC, and national strategic plans related to universal health coverage. Specifically, Standalone national PC policies were identified in 14 countries (1, 6, 17, 22, 25–33) and formal national cancer or Human Immunodeficiency Virus (HIV) Policies that included sections addressing PC were reported in 35 of the 54 African countries (65%) (11, 17, 34, 35, 45, 55, 65, 68, 69), and Equatorial Guinea (70), Eritrea (71), Namibia (72), Sudan (11), and Seychelles (73) included PC in their national strategic plans related to universal health coverage. Only Rwanda and South Africa report formal policies on the integration of PC in their primary health care systems, but Uganda is working toward this (6).

**Table 5:** PC health policies in African countries.

Stand alone PC national plan (*n* = 14/54; 26%)	PC in national cancer plan (17) (*n* = 23/54; 43%)	PC in national HIV plan (17) (*n* = 23/54; 43%)	PC included in national health plan/strategy (*n* = 5/54; 9%)
Benin (30) Botswana (26, 31) Cape Verde (32) Eswatini (27, 45) Ethiopia (17) Guinea (17) Kenya (6, 25) Libya (11) Malawi (17, 67) Mozambique (33, 45) Rwanda (17, 28) South Africa (6, 37) Tanzania (6) Zimbabwe (1)	Algeria (35) Angola^a^ (11) Botswana Burkina Faso Cameroon^a^ (55) Côte d'Ivoire Egypt (34) Eswatini Ghana Guinea Kenya Liberia^a^ (65) Libya^a^ (11) Madagascar^a^ (11) Mauritius Morocco (35) Namibia Rwanda Senegal Sudan Tanzania Tunisia (35) Zambia	Botswana Burkina Faso Central African Rep. Côte d'Ivoire Eritrea Eswatini Ethiopia Gambia Ghana Guinea Kenya Libya^a^ (11) Malawi (68) Mali Mozambique^a^ (45) Namibia Nigeria (69) Sierra Leone South Africa Tanzania Uganda Zambia Zimbabwe (45)	Equatorial Guinea (70) Eritrea (71) Namibia (72) Sudan (11) Seychelles (73)

Modalities of Palliative Care (PC) integrated into Health Policies across Africa.

^a^ Countries in which the data source differs from the column heading.

A formal PC office or desk was identified in the Ministry of Health of 21 countries (6, 17, 34). A national budget for PC is also mentioned in 13 countries: Egypt (36), Libya (11), Algeria, Botswana, Côte d'Ivoire, Eswatini, Ethiopia, Gambia, Malawi, Mauritius, Namibia, Rwanda, Uganda (17). In some countries such as Eswatini and Uganda most of the funds for PC services come from external donors (2, 27) and faith-based organisations (38). In Rwanda (6, 19) and South Africa (6), health insurance covers PC.

### Research

National research groups on PC were only described in Morocco (34, 35), South Africa (6) and Uganda (22). However, efforts to build research capacity were reported in Cameroon through the offering of Master's and PhD programs with a focus on PC research for clinical professionals (55) and articles on PC development were retrieved from the databases for 29 countries (Table 6). National PC congress or conferences were reported in 8 countries: Cameroon, Democratic Republic of Congo, Côte d'Ivoire, Gambia, Kenya, Nigeria, South Africa and Uganda (17).

**Table 6:** The number of PC development articles by country.

Country	Number of PC development articles retrieved	Country	Number of PC development articles retrieved
Uganda	22	Botswana	3
South Africa	16	DR Congo	3
Egypt	15	Morocco	3
Malawi	15	Senegal	3
Kenya	14	Sudan	3
Nigeria	10	Tanzania	3
Rwanda	8	Zimbabwe	3
Mozambique	6	Gambia, The	2
Ghana	6	Namibia	2
Eswatini	5	Tunisia	2
Cameroon	4	Algeria	1
Ethiopia	4	Burundi	1
Zambia	4	Côte d’Ivoire	1
Liberia	4	Lesotho	1
		South Sudan	1

Twenty-nine countries (54%) have published articles related to Palliative Care development from 2017 to 2023 showing an increasing activity related to African PC research.

### Education and training

PC is recognized as a speciality in Kenya by the Kenya Medical Board (74), and as a sub-speciality of Family Medicine in Ghana (38). Despite this, there are no residency or fellowship training programs in Kenya (74). Regarding PC in the undergraduate curricula, 24 countries integrated PC into medical schools and 22, into nursing schools (11, 17, 31, 34, 36, 37). At the post-graduation level, eight countries (Egypt, Ghana, Kenya, Malawi, Rwanda, South Africa, Tanzania and Uganda) reported programs in PC that vary in length and modality (1, 11, 25, 28, 38–44). Five of them, Ghana (38), Kenya (22, 43, 74), Rwanda (28), South Africa (42), and Uganda (25, 41) have programs for nurses. Some of those courses also train clinical officers and midwives in Ghana (38) and Uganda (41). In addition, Botswana (31), Ethiopia (75), Gabon (11) and Rwanda (1) are in the process of developing PC post-graduate certification.

Eleven countries depended on external international professionals or institutions to provide PC training (11, 26, 32, 39, 40, 46, 63, 68, 76). Uganda-based professionals were often involved in these (11, 26, 39, 40, 63).

Paediatric PC education programs were also reported in Kenya, Tanzania (1), South Africa (37) and Uganda (41). In addition, 15 French-speaking African countries were involved in a three-year project to train professionals in paediatric PC (76).

New training through short courses was described in several countries that previously had not reported these. These included Sudan (11), Sao Tome and Principe (24) and Cape Verde (32). Distance learning programs were reported in Kenya (22), Nigeria (77) and Uganda (25, 74).

Some PC training initiatives extended beyond nursing and medicine. These included: community volunteer workers in Kenya (6), Malawi (66), Rwanda (28) and Uganda (25); pharmacists in Malawi (39) and Kenya (25); and spiritual caregivers, traditional healers, and allied health professionals in Uganda (22, 25).

### Essential medicines

Morphine availability was reported in 28 countries (52%) (6, 17, 22, 25, 36, 45–48). Yet, in Sudan, opioids are reserved exclusively for cancer patients (46) and 50% of those countries stated inconsistent availability and shortage of supply chain issues related to morphine (26, 34, 36, 39, 45–47, 65, 78, 79).

Regarding national production, oral morphine solution is produced in Eswatini (49), Kenya (25), Malawi (49), Rwanda (19), Sierra Leone (11), Tanzania (6) and Uganda (22). However, oral morphine availability is often restricted to hospitals and large cities (45, 46) except for Rwanda (80) and Uganda (46). In the latter two countries, national morphine production enabled access to opioids in primary care (80) and the community- and village-settings (46). Additionally, Rwanda has implemented legislation to ensure the security of the supply of morphine (19).

There are many barriers related to accessing essential medicines. Barriers to prescribing opioids were described in 28 countries. In Eswatini (45), Ethiopia and Tanzania (6), for example, physicians are required to obtain a special licence or authorization to prescribe opioids. However, in Cameroon, Eswatini, Kenya, Malawi, Malí, Uganda, Sierra Leone, Tanzania, Zimbabwe (17), and Rwanda (29) nurses are allowed to prescribe morphine. In Uganda, midwives are authorised to issue prescriptions for pethidine to women in labour (50).

Other barriers that have been mentioned include myths and misconceptions related to opioid analgesics, restrictive legislation (31, 33, 38, 46), affordability issues (33, 45, 47, 48, 81), geographical distance (45, 48, 81), insufficient training (22, 48), prescriber shortages (45); legal limitations on opioid dosage per prescription (46) and insufficient funding (82). To address the problem of poor affordability, four countries have implemented solutions. Malawi and Tanzania (47) have introduced subsidies, while Zimbabwe (45) and Uganda (22, 25, 50, 81) provide morphine for free in the public sector.

Despite the increase of national morphine supply and consumption in a few countries such as Senegal (78) and Rwanda (19, 23), the mean and median opioid consumption of morphine across Africa remains extremely low (<3 mg/capita/year) (11). The only exceptions are Tunisia with 3,99 mg/capita year, Mauritius with 4,75 mg/capita/year, Eswatini with 5,17 mg/capita/year and South Africa with 13,24 mg/capita/year (11). Twenty per cent of African countries, namely Algeria (11), Ghana (83), Kenya (22), Mozambique (84), Nigeria (83), Rwanda (6), Senegal (78), South Africa (79), Tanzania (47), Uganda (81) and Zambia (20) had articles explaining the use of other medications included in the WHO PC Essential Medicines List. Kenya reported the inclusion of 14 PC medications by the Kenyan Government, a relatively high number (22). For Rwanda, PC medicines were available in district pharmacies (6).

### Service provision and implementation

**Figure 2:**
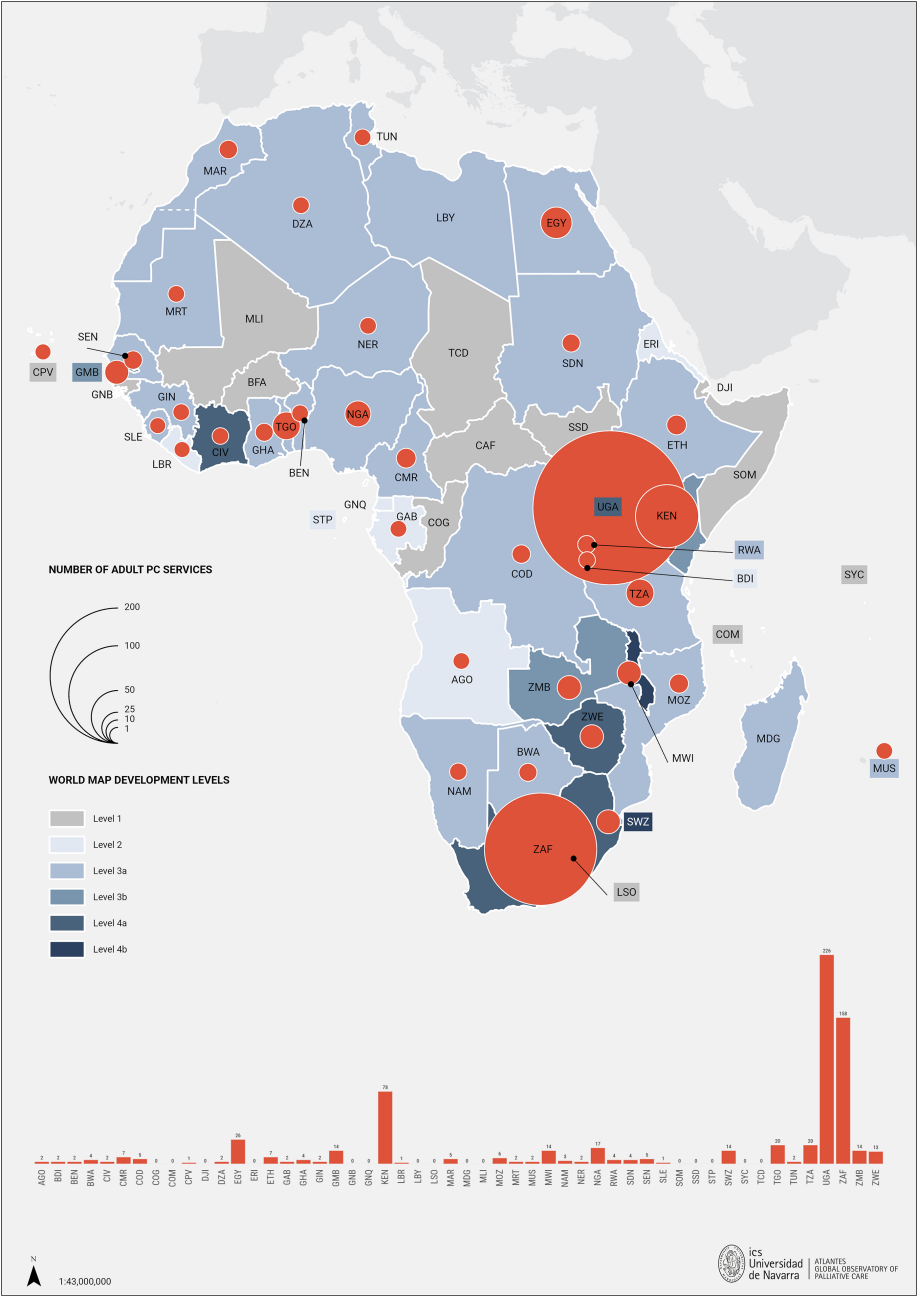
The number of palliative care services across African countries and their corresponding levels of development. AGO, Angola; BDI, Burundi; BEN, Benin; BFA, Burkina Faso; BWA, Botswana; CAF, Central African Republic; CIV, Republic of Côte d'Ivoire; CMR, Cameroon; COD, Democratic Republic of the Congo; COG, Congo Republic; COM, Comoros; CPV, Cabo Verde; DJI, Djibouti; DZA, Algeria; EGY, Egypt; ERI, Eritrea; ETH, Ethiopia; GAB, Gabon; GHA, Ghana; GIN, Guinea; GMB, Gambia; GNB, Guinea Bissau; GNQ, Equatorial Guinea; KEN, Kenya; LBR, Liberia; LBY, Libya; LSO, Lesotho; MAR, Morocco; MDG, Madagascar; MLI, Mali; MOZ, Mozambique; MRT, Mauritania; MUS, Mauritius; MWI, Malawi; NAM, Namibia; NER, Niger; NGA, Nigeria; RWA, Rwanda; SDN, Sudan; SEN, Senegal; SLE, Sierra Leone; SOM, Somalia; SSD, South Sudan; STP, Sao Tome y Príncipe; SWZ, Eswatini; SYC, Seychelles; TCD, Chad; TGO, Togo; TUN, Tunisia; TZA, Tanzania; UGA, Uganda; ZAF, South Africa; ZMB, Zambia; ZWE, Zimbabwe.

The search revealed that only 17 countries did not have PC services or had no publications describing them. Uganda has the highest number of PC services in Africa (*n* = 226) (2), followed by South Africa (*n* = 158) (6), Kenya (*n* = 78) (51), and Egypt (*n* = 26) (34). To illustrate these results in this review, a map, was adapted combining information on the latest level of PC development (85) with the number of services for each country (see Figure 2) (2, 6, 11, 17, 26, 31, 33, 34, 40, 42, 48, 51–54). Even though Uganda and Kenya have the highest number of services in the continent, only 10% of their population in need of PC can access it (25). More importantly, are the ratios of services by 100.000 population. In which, the previous top four would lose their position (as their ratios are, respectively: 0,36, 0,27, 0,15 and 0,02) to Eswatini and The Gambia (ratios, respectively: 1,17 and 0,53).

Paediatric PC services were reported in Botswana (26), Cameroon (55), Egypt (36), Eswatini, Ghana, Morocco, Nigeria, Senegal, Zambia (11), Kenya, Malawi, Tanzania, Uganda, Zimbabwe and South Africa (1). In the latter, the highest number of services were reported, a total of 40 (42). Service imbalances between rural and urban areas were reported in Botswana (31), Eswatini, Mozambique, Zimbabwe (45), Malawi (67), South Africa (86) and Uganda (46). However, innovative mobile phone (mHealth) interventions are being deployed and are currently under development to enhance the delivery of PC services across Africa (87).

## Discussion

This study aimed to describe the current status of PC development in Africa by scoping the literature in the last 6 years. A total of 118 new articles met the inclusion criteria, and some activities across the six domains of PC development were reported in 39 out of 54 African countries. Compared to the previous review in 2017 (1), there was a 140% increase in the number of publications and a 50% increase in the number of countries. This signals ongoing growth and interest in developing PC at national levels but also reveals regional variability. In this new approach, geographically, Central Africa is the least developed subregion. In contrast, Southern Africa is more advanced, with at least 60% of the countries showing development across all six dimensions. These disparities are influenced by economic, political, infrastructural, cultural, and educational factors (88).

Public awareness of what palliative care is remains very low. Misconceptions abound, including among health professionals (22, 26, 27, 34, 39, 55, 59, 60) “(…) main assumption was that when you’re referring a patient for palliative care, there's nothing more that can be done for the patient” (89). Strategies to address these appear to be growing (6, 22, 23). National PC associations play important advocacy roles in promoting PC in their respective countries., and since 2017, the number has more than doubled, as reported in 26 countries (1, 6, 11, 17).

Regarding health policies, the number of countries with a stand-alone national strategy or plan for PC has more than doubled, reaching a total of 14 (1, 6, 11, 17, 25–27, 30–33, 37, 45). However, these countries remain scattered examples across the continent. Central Africa, for instance, is the least developed subregion in this regard, with no country yet establishing a standalone PC policy. No less important, Benin recently highlighted the effectiveness of these implemented policies should be systematically and regularly evaluated (90).

In addition, despite limited PC development, some countries, such as South Sudan, have included essential PC components – such as sharing information and communicating about prognosis, goals of care, and decision-making guidance – in their COVID-19 guidelines (58). That raised the hypothesis that the COVID-19 pandemic may have acted as a catalyst in PC.

Despite the rise in palliative care publications across Africa, there is a clear imbalance, with over 80% of the articles in this review coming from Anglophone countries, highlighting language inequities in scholarly publishing (1, 21, 91). In the Southern Africa subregion, all countries published at least one article on PC development in the last 6 years. Although only three countries reported a dedicated PC research group (6, 22, 34, 35), regional groups are working toward advancing the research agenda and creating a critical mass of researchers across Africa, such as the APCA African Palliative Care Research Network (92) and the African Centre for Research on End of Life Care (ACREOL) (93).

An encouraging finding is a significant increase in PC education across the continent; this has more than doubled since the last review (1), and 29 countries have PC integrated into undergraduate or postgraduate education in medical or nursing schools (11, 17, 31, 34, 36, 37). Collaborations with academics, professionals, and institutions from other countries contributed to palliative care education and training in Malawi. However, it is recognised that enrolment in and completion of accredited training programs often depend on the training's potential to enhance career prospects: “For people to do palliative care training, that doesn't advance their career and for interest, and that… no one's done that.” (89). Short courses on core palliative care skills are now reported in countries where PC has been incipient, including Cape Verde (32), Liberia (63), Sudan (11) and, Sao Tome and Principe (24). Technology plays an important role in education, particularly in enhancing engagement and instructional practices. For instance, in Malawi, Uganda and Botswana, an online course has successfully enhanced paediatric Palliative care education (89).

An important pillar of PC development has always been the use of opioids. There has been an increase in morphine supply (available in 28 countries) since the last review (1). To overcome some barriers to access to opioids, some countries invested in morphine powder and national production of morphine solutions has increased twofold across the continent (6, 19, 22, 49, 50). Another strategy is related to the lack of prescribers mass, in which 19% of the countries allow nurses to prescribe opioid analgesics (6, 17, 25, 29, 46, 50). However, availability and accessibility remain restricted in half of African countries as per published reports, and the median consumption remains under 3 mg/per capita/year in general, except for Tunisia, Mauritius, Eswatini and South Africa (4, 9–13, 57).Unfortunately, Africa's median opioid consumption­ represents 1% of the minimum recommended by experts (94) and remains the lowest worldwide. This is particularly concerning given the demonstrated high PC needs across Africa relative to other world regions (85).

In this review, a total of 675 adult specialised PC services were identified across the continent [317 more services than in 2017 (1)], and paediatric services are now available in 15 countries (nine more countries than in 2017). In Egypt, the number of services rose from 3 (1) to 26 (34), and in Uganda, from 34 (1) to 226 (2) accredited PC facilities. However, the data confirms ongoing gaps, insufficient access and uneven distribution of PC across the continent. These gaps were already evident in previous reviews (1, 85). About 70% of all services in the continent are concentrated in only three countries: Uganda, Kenya and South Africa. Also, as highlighted by Kagarmanova et al, in Uganda, high coverage of palliative care facilities does not necessarily equate to patient access, as barriers like transportation difficulties and high medical costs can restrict availability and accessibility (2). However, the use of mHealth - defined as the use of mobile wireless technologies for health— in palliative cancer care in Uganda is helping to overcome some of these barriers by improving the frequency and ease of communication with patients, supporting remote symptom management, and facilitating quicker decision-making, ultimately enhancing patient comfort and care (87). The ratios of services per population range from highs of 1,17, 0,53 and 0,36 in Eswatini, Gambia, and Uganda, respectively, to under 0,10 services per 100.000 inhabitants in the majority of countries (*n* = 27).

Only Rwanda and South Africa have formally integrated PC into the primary healthcare systems, through a National Strategic Plan for PC. Rwanda's government, supported by Rwanda Palliative Care & Hospice Organization, devised and tested a model for integrating coordinated PC throughout all levels of the public healthcare system and initiated training of new personnel of home-based practitioners to deliver PC within residences. In 2019, the South African parliament proposed legislation for a National Health Insurance bill, incorporating an extensive package of PC services tailored for primary care settings (6). National palliative care policy aimed to establish a costing formula by 2018, but this has yet to be achieved: “Because they feel like palliative care is not a healthcare priority.” (88). Throughout the continent, there continues to be a substantial disparity in access to PC services between rural and urban regions (31, 45, 46, 67, 86). Challenges in implementing complex interventions, such as integrating palliative care in the health and educational system, may stem from factors beyond limited resources, such as organisational culture, leadership, social support, and readiness for change. Additionally, these same contextual factors may influence the scarcity of financial resources. Elements like patient profiles, motivation, vision, education, and underlying assumptions further contribute to the complexity of the context (88).

Since the publication by Rhee and colleagues in 2017 (1), this is the most extensive literature review and provides a useful update on the current PC situation. In addition to the approaches used by Rhee et al., we strengthened the search by broadening the criteria to include any language and by using the “specific filter” guidelines suggested by Rietjens et al. (16) We also used the most recent WHO domains on PC development, which adds two additional domains to the four used previously.

This review has several limitations. First and foremost, the absence of a publication reporting on one or other aspect of PC development in a country does not necessarily mean the absence of such activity in that country. Second, potential biases include publication bias, as the search was limited to three databases, potentially excluding relevant studies published elsewhere. It is possible that other reports on PC activities may have been missed, especially since challenges of health services organisation and delivery in Africa are often approached in the context of broader global health issues. Third, publication delays may have resulted in some activities being present in a country but not yet reported on. Fourth, we did not undertake a comprehensive search of the grey literature, including an in-depth and broad search of websites. Moreover, our search on official sources, such as government websites, was restricted to countries with two or fewer articles retrieved from the formal databases. This means that in 20 countries this source was not accessed and may have resulted in us missing some activities. Lastly, there was considerable variability in the quality of the reports included in our review, although a formal analysis of the quality of each article was outside the scope of this review. We anticipate that the use of a rigorous search approach, credible databases, and restriction to peer-reviewed journals helped mitigate this limitation. Therefore, it is possible that this review missed some PC development activities occurring across the continent.

## Conclusions

Our findings show that, despite all the challenges faced in Africa in the last few years, growth in the development of PC across several domains has been reported across the continent since the last report in 2017. This is encouraging and demonstrates some movement since the resolution passed by the World Health Assembly in 2014 calling on all member states to integrate palliative care into their healthcare systems. However, many gaps remain and a considerable amount of effort by policymakers, elected officials, funders, health care professionals and communities across all levels of health care delivery, government, health services organisation and communities are still needed.

Policymakers should prioritise integrating palliative care into national health strategies, and healthcare providers need ongoing education in palliative care to effectively address patient needs. Continuous monitoring and evaluation are crucial to ensure sustained progress in African palliative care development. We expect more changes in this direction, as integrating PC in all levels of healthcare systems is part of universal health coverage.

Funders, including international agencies and local governments, play a vital role in sustaining palliative care (PC) infrastructure. Their investments in research, ongoing education, and training are fundamental to developing a sustainable PC workforce and ensuring effective, accessible care. In particular, prioritizing support for Francophone, Lusophone, and Arabic-speaking countries—often facing more significant development challenges—can foster a more equitable and consistent distribution of PC services across the continent.

Leveraging the deep-rooted communal values within African cultures—embodied in concepts like Ubuntu (95)—creates a powerful, culturally aligned foundation for advancing PC. Local support systems can extend culturally sensitive PC to underserved areas and offer essential support to patients and families by engaging communities in caregiving, raising awareness, and forming volunteer networks.

Future research should incorporate other sources of information to ensure optimal coverage. These should include a comprehensive analysis of national policies and strategies, official reports from ministries of health, and online publications from advocacy and groups. In addition, the inclusion of global health databases such as African Index Medicus and publications from WHO, APCA and Human Rights Watch should be considered as their documents are usually not indexed in the databases used and may add relevant information on this topic. In addition, future research could benefit from a deeper exploration of how geographical, cultural and socio-economic factors influence the development and implementation of PC services. Moreover, incorporating the perspectives of patients receiving PC and their families could provide valuable insights for further development.
