# Supplementary material for: Comprehensive scoping review of palliative care development in Africa: recent advances and persistent gaps
Source: Front Health Serv. 2024 Dec 9;4:1425353. doi: 10.3389/frhs.2024.1425353 (PMC11663863; doi:10.3389/frhs.2024.1425353)
Supplement: Supplementary file 1 [file Table1.docx]

**Supplementary Material**

**Table Supplementary 1. WHO PC development dimensions and the corresponding information collected**

WHO - World Health Organization, PC - Palliative Care, HIV - Human Immunodeficiency Virus.

| **EMPOWERING PEOPLE AND COMMUNITIES** | - Groups promoting the rights of PC patients and families |
| --- | --- |
|  | - Advance directives |
|  | - Profesional cooperation |
|  | - Awareness |
|  | - Volunteers |
|  | - Others related |
| **POLICIES** | - National Strategy |
|  | - HIV/Cancer plan |
|  | - PC in primary health |
|  | - National authority in the Ministry of Health |
|  | - Regulations |
|  | - Funding |
|  | - Others Policy-related |
| **RESEARCH** | - National PC congress |
|  | - Number of PC articles |
|  | - Research PC group in the country |
|  | - Others research-related |
| **EDUCATION** | - Specialisation |
|  | - Undergraduate education |
|  | - Other education (diplomas, professors, continuous edu) |
| **PAIN MEDICINES** | - Opioids Consumption |
|  | - Essential medicines at all levels of care |
|  | - Oral morphine at the primary level |
|  | - Availability |
|  | - Prescription |
|  | - Affordability |
|  | - Barriers (regulations, opiophobia) |
| **SERVICES** | - Adult Services |
|  | - Pediatric Services |
|  | - PC provision at the primary level |
|  | - Others service-related info |
